# Suppression of* IL-6* Gene by shRNA Augments Gemcitabine Chemosensitization in Pancreatic Adenocarcinoma Cells

**DOI:** 10.1155/2018/3195025

**Published:** 2018-03-06

**Authors:** Hai-Bo Xing, Meng-Ting Tong, Jing Wang, Hong Hu, Chong-Ya Zhai, Chang-Xin Huang, Da Li

**Affiliations:** ^1^Department of ICU, Sir Run Run Shaw Hospital, School of Medicine, Zhejiang University, Hangzhou, China; ^2^Department of Medical Oncology, Sir Run Run Shaw Hospital, School of Medicine, Zhejiang University, Hangzhou, China; ^3^Department of Medical Oncology, The Affiliated Hospital of Hangzhou Normal University, Zhejiang University, Hangzhou, China

## Abstract

Pancreatic adenocarcinoma has an exceedingly poor prognosis, accounting for five-year survival of less than 5%. Presently, improving the efficacy of pancreatic adenocarcinoma treatment has been the focus of medical researchers worldwide. Recently, it has been suggested that deregulation of interleukin- (IL-) 6 is caused by a key gene involved in the beginning and development of pancreatic adenocarcinoma. Herein, we investigated whether suppression of IL-6 could augment gemcitabine sensitivity in the PANC-1 cells. We found considerably higher expression of IL-6 in pancreatic adenocarcinoma tissues than that in the adjacent nontumorous tissues. Suppression of IL-6 by shRNA resulted in apoptosis as well as inhibition of cell proliferation and tumorigenicity. In addition, suppression of IL-6 remarkably promoted antitumor effect of gemcitabine, indicating that the combination of shRNA targeting IL-6 with gemcitabine may provide a potential clinical approach for pancreatic cancer therapy.

## 1. Introduction

Pancreatic adenocarcinoma is a type of malignant tumor characterized by an extremely low 5-year survival of 5% or less [1, 2]. Local invasion and lymphatic metastasis occur in 80% of the clinical cases, and their prognosis of disease progression is poor [3, 4]. While radiotherapy is one of the most important therapeutic methods for pancreatic adenocarcinoma, chemotherapy with gemcitabine following tumor surgery can contribute remarkably to a substantial delay of relapse. After absorption by cells, gemcitabine is phosphorated by deoxycytidine kinase to form dFdCTP, which competes with dCTP for insertion into the deoxycytidine sites of DNA strands, by which mechanism it destructs DNA replication and consequently results in cell death. However, the functional mechanism of gemcitabine is still obscure, and further studies are necessary to elucidate the mechanism of gemcitabine resistance.

Interleukin-6 (IL-6), a cytokine with pleiotropic function, is synthesized by various types of cells, such as endothelial cells, macrophages, myeloid cells, and fibroblasts. It promotes cell proliferation and differentiation, participates in the immune defense system, and is involved in almost all physiological processes. Moreover, the involvement of IL-6 is critically important in the development of various conditions, such as infections, traumas, and hematopoiesis [5–8]. The expression of IL-6 has been found to increase dramatically in cases of a number of different cancer types, including liver, colon, and ovarian cancer [9–11]. In pancreatic adenocarcinoma patients, IL-6 upregulates other cytokine expression and is involved in proliferation, resistance to apoptosis, and immune evasion [12]. IL-6 becomes bound to a composite receptor consisting of a distinct 80 kDa alpha subunit (IL-6R*α*, gp80) and two gp130 receptor subunits transducing a signal after the ligand binding. Then, gp130 triggers the activation of Janus kinase (JAK2)/signal transducer and activator of transcription 3 (STAT3) pathway through the induction of cross-phosphorylation of the JAK2 peptides linked to the cytoplasmic side of the receptor [13, 14]. STAT3 is an important member of the STAT family that is constitutively induced in a broad spectrum of human malignant cancers [15] and is recognized as a major factor for oncogenesis in many epithelial malignant tumors. STAT3 is also tightly involved in the development of skin and gastric cancers in mouse models [16, 17]. Considering the substantially important roles of STAT3 in the development and progression of a large number of cancer types, we speculated that blocking IL-6 expression in STAT3 signaling may be valuable in the treatment of pancreatic adenocarcinoma.

In this study, we demonstrated that suppression of IL-6 expression resulted in inhibition of* in vitro* and* in vivo* tumorigenicity of the pancreatic adenocarcinoma cells. In addition, knockdown of IL-6 expression sensitized pancreatic adenocarcinoma cells to gemcitabine. These findings not only revealed the vital role IL-6 exerts in pancreatic adenocarcinoma development, but also highlighted that the combination of gemcitabine and shRNA targeting IL-6 can potentially be applied in clinical practice for the treatment of pancreatic adenocarcinoma.

## 2. Materials and Methods

### 2.1. Plasmids and Cell Culture

IL-6 shRNA expressing plasmids were designed and constructed by Genechem Corporation (Shanghai, China). The sequence of shRNA-IL-6 was “gacactattttaattatttttaa.”

Dulbecco's modified Eagle's medium (DMEM)/F12 supplemented with 10% fetal bovine serum (FBS) was used for culturing all cell lines. To suppress IL-6 expression, a plasmid containing IL-6-shRNA was transfected into the PANC-1 cells, and the positive clones were identified using puromycin (300 ng/mL) for a period of 24 h.

### 2.2. MTT Assay

Effect of shIL-6, gemcitabine, or shIL-6 transfection plus gemcitabine treatment on the proliferation of PANC-1 cells was determined by MTT assay. Briefly, a volume of 20 *μ*L MTT was applied to each well of a 96-well plate and incubated with the cells at 37°C for 4 h. Next, 150 *μ*L/well DMSO was admixed to obtain coloration, and samples were shaken gently for 2 h at room temperature in the dark. To measure cell viability, the values of absorbance at 490 nm were determined. Data from triplicate wells per treatment were collected and the experiments were repeated three times at separate occasion.

### 2.3. Assessment of Colony Formation

The transfected PANC-1 cells were transferred into a six-well plate and were cultured in DMEM with fetal bovine serum (10%). After 24 h, the medium was replaced with fresh medium containing G418 (400 mg/mL) and changed every other day. After 14-day incubation, cells were fixed with methanol and stained with 0.1% crystal violet. The number of visible colonies was counted.

### 2.4. Evaluation of Cell Invasion

Assessment of cell invasion was conducted with the Boyden chambers using coated Matrigel following the instruction provided by the manufacturer (BD Biosciences, San Jose, CA). Staining of the invasive cancer cells was performed with crystal violet, and they were observed and counted under a microscope. The experiments were performed twice in triplicate at minimum.

### 2.5. Flow Cytometry Analysis

Detection of cellular apoptosis was done by double staining of Annexin V and propidium iodide (PI) following the previously published method [18]. Trypsin (0.25%) was utilized to harvest the cells, followed by double washing with PBS. Further, the cells were resuspended in binding buffer (250 *μ*L) that was calibrated to 1 × 10^6^/mL. Then, to the cell suspension, the staining solution that contained Annexin V-FITC and PI was added followed by incubation for 30 min in the dark. Apoptosis was then analyzed by flow cytometry using FACSAria System (Becton Dickinson, Franklin Lakes, NJ, USA).

### 2.6. Quantitative Real Time RT-PCR

Total RNA was extracted with the RNeasy Mini kit (Qiagen) following the manufacturer's guidelines. The specific experiments were conducted with SYBR Green Power Master Mix complying with the instructions provided by the manufacturer (Applied Biosystems). Reverse transcription of the reaction mixture (20 *μ*L) containing total RNA (1 *μ*g) to cDNA was done with PrimeScript RT-polymerase (Life, Shanghai, China). Then, the cDNA was used for real time quantitative PCR utilizing primers (Generay, Shanghai, China) distinct for STAT3, JAK2, Bcl-2, Bax, caspase-3, and caspase-9. GAPDH was used as an internal control. All reactions were carried out on the Applied Biosystems 7500 Sequence Detection System (Applied Biosystems, Foster City, CA, USA). The levels of relative expression were calculated as ratios normalized against those of GAPDH. Comparative quantification was performed using the 2^−ΔΔCt^ method. Sequences of the primers were listed in Table 1.

### 2.7. Immunoblotting

Denaturing SDS-PAGE sample buffer was utilized for PANC-1 cell lysis through standard methods. Then, separation of protein lysates was done with 10% SDS-PAGE, and they were transferred onto nitrocellulose membranes. TBS containing 0.1% Triton X-100 and 5% nonfat milk was used overnight at 4°C to block the membranes, followed by incubation at 4°C overnight with primary antibodies of STAT3, p-STAT3, JAK, p-JAK, Bcl-2, Bax, caspase-3, caspase-9, and *β*-actin (Santa Cruz Biotech, Santa Cruz, CA, USA). Upon washing, the membranes were subjected to incubation with HRP-conjugated secondary antibodies for 2 h at room temperature. Detection of the signal was accomplished with the ECL reagents.

### 2.8. Animal Experiments

The animal experiments were conducted in compliance with the Guide for the Care and Use of Laboratory Animals, and the study protocol was approved by the Animal Ethics Committee of Zhejiang University. Injection of 1 × 10^7^ cells in 100 *μ*L PBS was subcutaneously administered in the right back area of nude mice. Then, upon reaching tumor volume of approximately 150 mm^3^, the animals were randomly divided into groups (20 animals each group) of nontreated control (NC), intraperitoneally injected gemcitabine alone at a dose of 100 mg/kg daily, injection of shIL-6-plasmid, or injection of shIL-6 plus gemcitabine. Indicators of drug toxicity, including behavior shifts, loss of weight, and changed feeding patterns were incessantly monitored during the entire trial. The tumors were collected from 4 animals of each treatment group and weighed at weeks 1, 2, 3, 4, and 5, and the tumor masses were established.

### 2.9. Immunohistochemical Staining

Streptavidin-peroxidase (SP) staining was performed for protein detection after retrieval of the antigens through microwave treatment. After inhibition of the activity of endogenous peroxidase by incubation in 3% H_2_O_2_ for 10 min, the samples were washed with PBS and incubated with anti-p-STAT3 or p-JAK2 antibodies at 4°C overnight (PBS served as a negative control). After coculture of the specimens with the respective secondary antibodies for 30 min, the specimens were subjected to streptavidin-peroxidase treatment for additional 30 min. After rinsing with PBS, diaminobenzidine (DAB) solution was applied. Next, counterstaining with hematoxylin was performed.

### 2.10. Statistical Analysis

SPSS15.0 software was used for the general statistical analysis. Student's *t*-test, one-way analysis of variance (ANOVA), *χ*^2^ test, or Wilcoxon test was employed to appropriately assess the significance between groups. All tests performed were two-sided, and *P* < 0.05 was considered statistically significant.

## 3. Results

### 3.1. Elevated IL-6 Expression Was Associated with Pancreatic Adenocarcinoma

**Figure 1:**
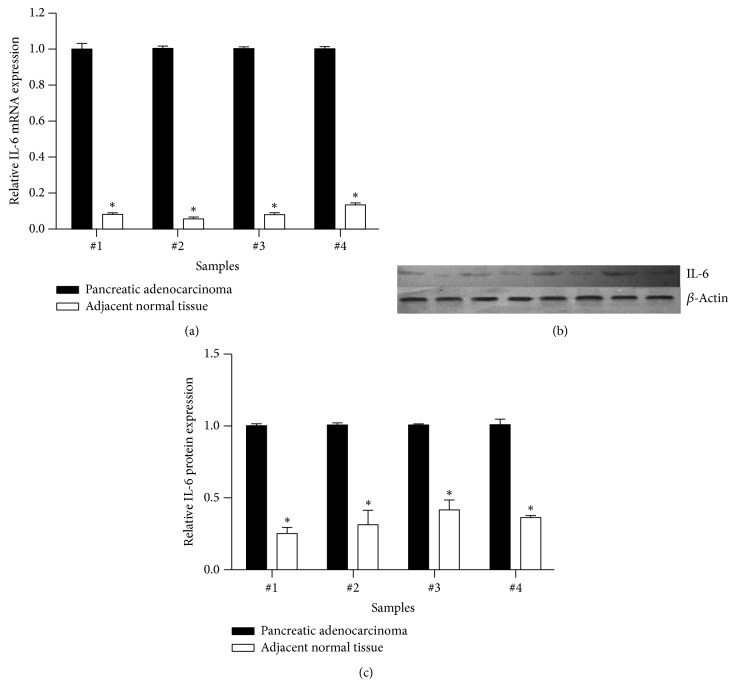
Expression of IL-6 mRNA and protein in four pancreatic adenocarcinoma tissues and their adjacent nontumorous tissues. Expression of IL-6 mRNA was quantified by real time RT-PCR and protein was semiquantitatively assessed by immunoblotting as described in the methods. ^*∗*^*P* < 0.05 compared with pancreatic adenocarcinoma.

Recent studies indicated the existence of a correlation between IL-6 expression and cancer cell. To identify the association of IL-6 expression and the development of pancreatic adenocarcinoma, by using real time RT-PCR and immunoblot, we compared the IL-6 mRNA and protein levels in pancreatic adenocarcinoma with that in their adjacent nontumor tissues. We found that the IL-6 expression in the pancreatic adenocarcinoma was significantly higher than that in the adjacent nontumor tissues (Figures 1(a), 1(b), and 1(c)).

### 3.2. Suppression of IL-6 and Its Effect on Gemcitabine-Mediated Antiproliferation and Apoptosis Induction

**Figure 2:**
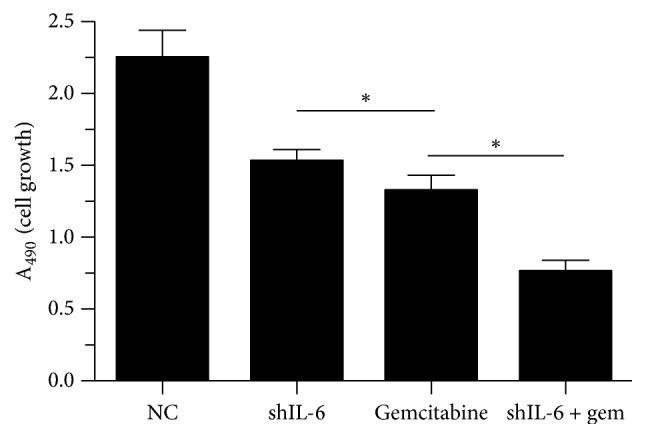
Effect of shIL-6 transfection and gemcitabine treatment on proliferation of PANC-1 cells. MTT assay was performed in the PANC-1 cells 48 h after shIL-6 transfection followed by treatment with or without gemcitabine. ^*∗*^*P* < 0.05.

**Figure 3:**
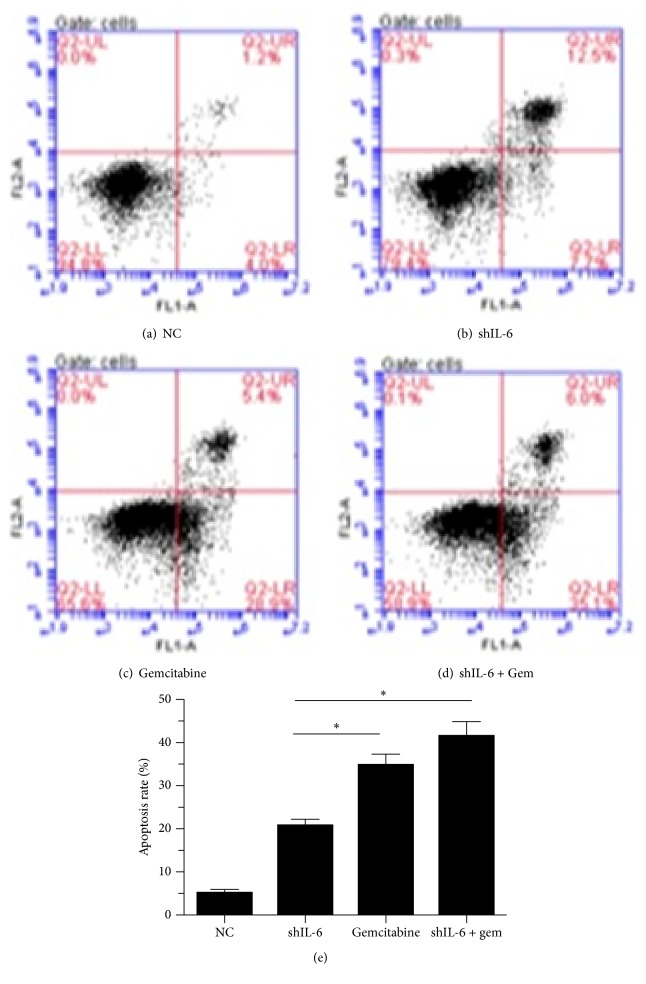
Effect of shIL-6 transfection and gemcitabine treatment on apoptosis of PANC-1 cells. Apoptosis assay was performed in the PANC-1 cells by flow cytometry analysis of Annexin V/PI staining 48 h after shIL-6 transfection followed by treatment with or without gemcitabine. ((a)–(d)) Representative results of flow cytometry analysis. (e) An average of 3 separate experiments, ^*∗*^*P* < 0.05.

To examine whether IL-6 affects tumor cell proliferation and apoptosis, we established stable transfectants with shIL-6 expression in PANC-1 cells. We found that IL-6 expression was significantly downregulated after 24 hours of transfection of shIL-6 into the cells. To determine the effect of shIL-6 on cell proliferation, the effects of the of shIL-6 alone or shIL-6 plus 100 *μ*M gemcitabine on the PANC-1 cells were examined by MTT assays. We observed that shIL-6 significantly inhibited PANC-1 cell proliferation in a time-dependent manner. Gemcitabine dramatically enhanced the inhibitory effect of shIL-6 (Figure 2). Furthermore, to evaluate the shIL-6-mediated promotion of apoptosis, PANC-1 cells with stably expressing control vector or shIL-6 and shIL-6 combined with gemcitabine were subjected to Annexin V analysis by flow cytometry. Suppression of IL-6 by shIL-6 alone resulted in significant apoptosis of the cells (20.2% versus 5,2% of control, Figure 3(b)), which was further augmented by the addition of gemcitabine (41.2%, Figure 3(d)).

### 3.3. Effect of IL-6 Suppression on Gemcitabine-Mediated Inhibition of Colony Formation and Cell Invasion

**Figure 4:**
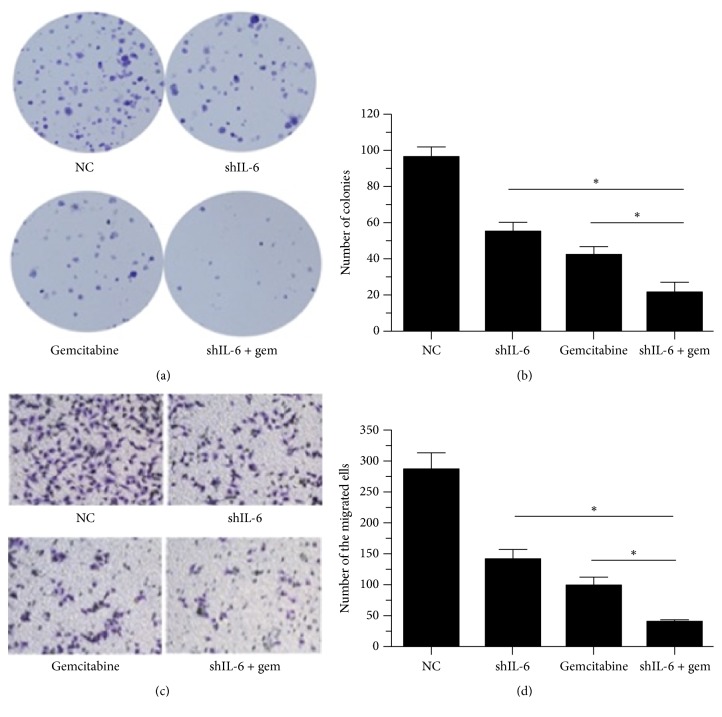
Effect of IL-6 suppression on colony formation and cell invasion. ((a) and (b)) Soft-agar assay was performed in the cells with stably expressing control vector or the shIL-6 and treated with or without 100 mg/kg gemcitabine. Data presented were from three independent experiments with duplicates in each experiment. ((c) and (d)) Invasiveness of the cells with stably expressing control vector or the shIL-6, treated with or without gemcitabine. Cellular invasive capability was determined with a modification of the Boyden chamber invasion assay as described in the Materials and Methods and representative images were presented in (c). The percentage of invasive cells from three separate experiments (mean ± SD) was presented as bar graphs (d). ^*∗*^*P* < 0.05.

We measured the* in vitro* tumorigenicity of the PANC-1 cells using a soft-agar assay and found that PANC-1 cells treated with gemcitabine alone or transfected with shIL-6 formed fewer colonies than the cells transfected with control vector. As expected, combination of shIL-6 transfection and gemcitabine treatment led to a more pronounced decrease in colony formation capacity compared to other groups (Figures 4(a) and 4(b)). To elucidate whether the knockdown of IL-6 could enhance gemcitabine-mediated tumor cell invasion, an invasion assay was performed. The results revealed that gemcitabine induced an approximately 3-fold decrease, that shIL-6 induced nearly a 2-fold reduction in cell invasion, and that the treatment with gemcitabine in the cells transfected with shIL-6 caused an even more remarkable decrease of cell invasion (7-fold) (Figures 4(c) and 4(d)).

### 3.4. Combined Effect of shIL-6 Transfection and Gemcitabine Treatment on Signal Transduction and Apoptosis-Associated Proteins

**Figure 5:**
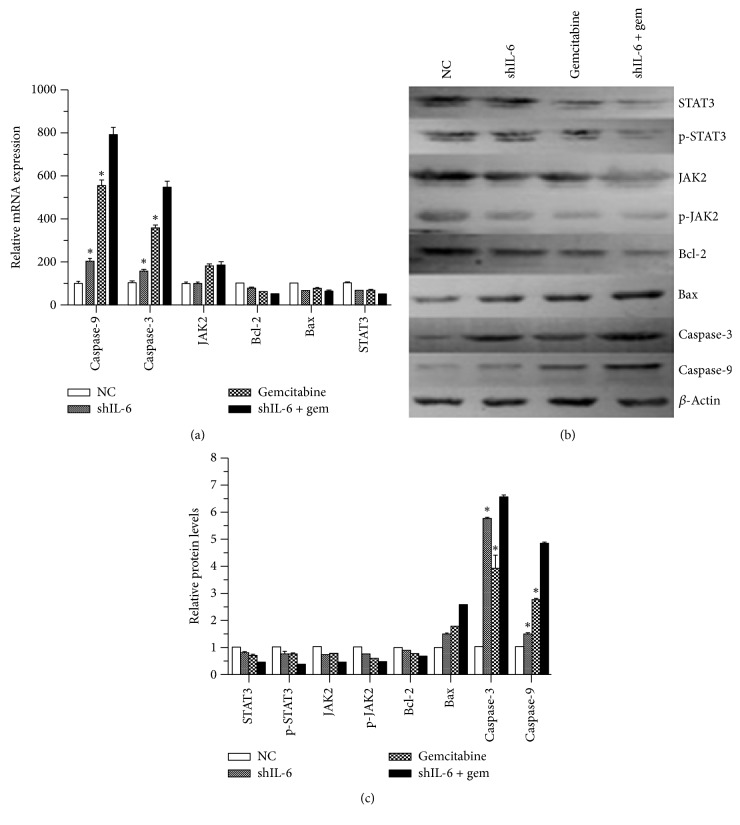
Effect of IL-6 suppression on STAT3 signaling and apoptosis-associated proteins. (a) Effect on the expression of caspase-3, caspase-9, IL-6, STAT3, JAK2, and Bcl2 mRNA. Expression of the indicated mRNA was quantified by real time RT-PCR in the cells after 48 hours of shIL-6 transfection followed by treatment with or without gemcitabine. ^*∗*^*P* < 0.05 compared with the nontreated control (NC). ((b) and (c)) Protein levels of STAT3, p-STAT3, JNK, p-JNK, Bcl-2, Bax, caspase-3, and caspase-9. Levels of the indicated proteins were semiquantitatively determined by immunoblot in the cells 48 hours after shIL-6 transfection followed by treatment with or without gemcitabine. Representative image data (b) as well as an average of three separate experiments (c) were presented. ^*∗*^*P* < 0.05 compared with shIL-6 + gemcitabine.

To elucidate the mechanism of the combination of shIL-6 and gemcitabine on apoptosis, expression of STAT3, p-STAT3, JAK2, p-JAK2 Bcl-2, Bax, caspase-3, and caspase-9 was investigated. After 24 h transfection of shIL-6, the PANC-1 cells were treated with or without 100 *μ*M gemcitabine. GAPDH or *β*-actin was utilized as an internal control. Real time RT-qPCR and Western blots results were performed in three separate experiments. Expression of the genes was normalized to the control in the respective experiment, and a value of 1.0 was assigned. In the group treated with the combination of shRNA-IL-6/gemcitabine (100 *μ*M), the expression levels of STAT3, p-STAT3, JAK, p-JAK, and Bcl-2 were substantially lower than those of the group with the single treatment. On the other hand, the expression levels of Bax, caspase-3, and caspase-9 in both the single treatment group and the group of the treatment with the shRNA-IL-6/gemcitabine (100 *μ*M) combination were higher than the ones in the control treatment (Figures 5(a), 5(b), and 5(c)).

### 3.5. The Combination of Gemcitabine and shIL-6 Enhanced an* In Vivo* Antitumor Effect

**Figure 6:**
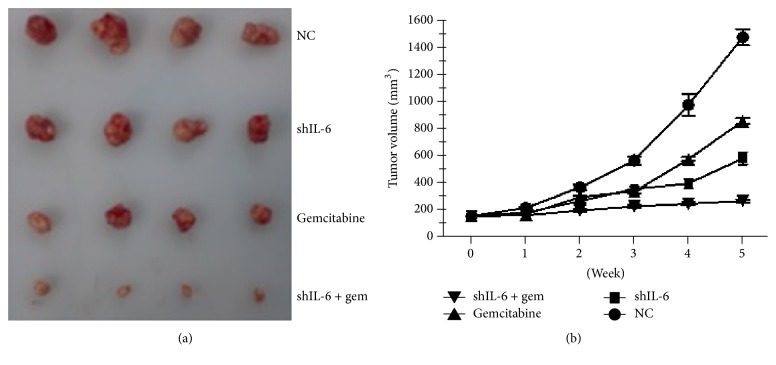
Antitumor activity of shIL-6 and gemcitabine in the xenograft model. Nude mice were randomly assigned to the following four groups: PANC-1 cells with stably expressing control vector, control vector with 100 mg/kg gemcitabine daily for 10 d via* i.p.* injection, shIL-6 alone, and shIL-6 plus 10 mg/kg gemcitabine daily for 10 d via* i.p.* injection. (a) Macrograph of the tumor tissues obtained on week 5. (b) Comparison of the tumor volumes as function of time.

To examine the* in vivo* tumorigenicity, we performed xenograft experiments, in which we administered injections in the right back area of nude mice with the following content: (1) PANC-1 cells with the control vector, (2) control vector with 10 mg/kg gemcitabine daily for 10 d via* i.p.* injection, (3) shIL-6, and (4) shIL-6 combined with 10 mg/kg gemcitabine daily for 10 d via* i.p.* injection. Mice were injected with 1 × 10^7^ of cells. As shown in Figure 6, gemcitabine or suppression of IL-6 caused a significant reduction in the size compared to the vector control group. Interestingly, the application of gemcitabine combined with shIL-6 resulted in an even more dramatic decrease of tumor size and weight (Figures 6(a) and 6(b)).

**Figure 7:**
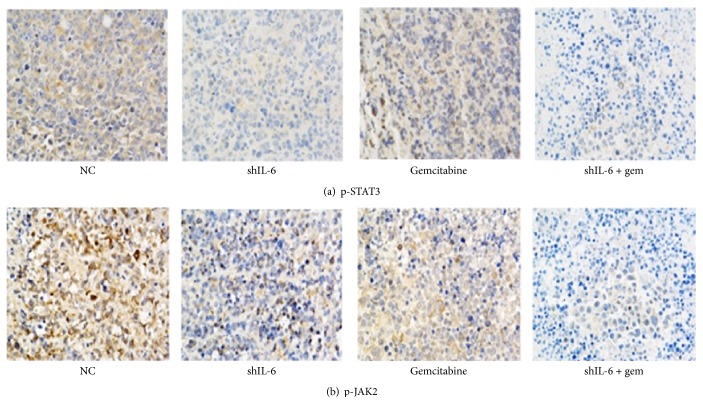
Representative images of p-STAT3 and p-JAK2 expression in the nude mice tumors assessed by immunohistochemistry. Magnification: ×200.

We then detected the protein expression of p-STAT3 and p-JAK2 in the different tumor groups by immunohistochemical staining. As illustrated in Figure 7, the immunostaining intensity of p-STAT3 and p-JAK2 in shRNA-IL-6 alone or gemcitabine (100 *μ*M) alone groups was lower than that in the control group, and the intensity was even lower in the treatment with the combination of shRNA-IL-6/gemcitabine (100 *μ*M).

## 4. Discussion

Many research results have evidenced that the one-year survival rate of pancreatic adenocarcinoma patients is less than 20%, and the five-year survival rate is under 5%. Moreover, wide cancer metastasis has happened in most diagnosed pancreatic adenocarcinoma patients. Eventually, the growth, metastasis, and invasion of cancer cells cause the death of the patients [19]. At present, in spite of the advances in surgical resection, radiotherapy, and chemotherapy, the contemporary therapeutic treatments have not succeeded in significantly improving the survival rate of patients with pancreatic adenocarcinoma [20]. Gemcitabine has been widely used in the treatment of pancreatic adenocarcinoma, but the acquired and intrinsic resistance of pancreatic adenocarcinoma cells can often lead to the need for repeated treatments [21]. The therapeutic targeting of pancreatic adenocarcinoma genes has attracted substantial scientific attention over the recent years [22]. The results of an experiment with mice revealed that the oncogenesis of melanoma and prostatic or gastric cancers could be inhibited by blocking of the IL-6/STAT3 signal pathway [23]. These findings indicated the potential application of interventions in the IL-6/STAT3 pathway for pancreatic adenocarcinoma therapy.

In our study, a shIL-6-plasmid was successfully constructed and used for transfection into the pancreatic adenocarcinoma cell strain PANC-1, achieving a silencing rate of more than 70%. The levels of protein and mRNA expression of STAT3 and p-STAT3 were considerably downregulated by interference with IL-6 expression. Meanwhile, we found that suppression of IL-6 induced apoptosis and reduced the cell survival or tumorigenicity of the cancer cells. These findings not only confirmed the vital role of IL-6 in development of pancreatic adenocarcinoma, but also highlighted that shRNA targeting IL-6 has the potential to become a powerful tool with its low toxicity, remarkable efficiency, and pronounced specificity.

Pancreatic adenocarcinoma cells have an acquired or intrinsic resistance to gemcitabine. In a previous examination, human pancreatic cancer cell lines (BxPc-3, PancTu-1, and Capan-1) were treated with sulfasalazine, an inhibitor of NF-K*β* gene, and it was found that sulfasalazine enhanced sensitivity of the cancer cells to chemotherapy [24]. Effect of the combination of gemcitabine and molecularly targeted agents, such as IL-6 inhibitor (shRNA), has been encouraging. In our study, proliferation of the cancer cells was substantially suppressed in a time-dependent pattern when gemcitabine and shRNA targeting IL-6 were used together. Moreover, this combination remarkably induced PANC-1 cell apoptosis. To better characterize the observed synergic effect, a tumor xenograft study was performed, in which shRNA targeting IL-6 and gemcitabine were used either alone or in combination. The combined treatment dramatically inhibited tumor growth compared to either treatment alone, suggesting that shRNA targeting IL-6 sensitized the xenografted pancreatic adenocarcinoma cells to gemcitabine.

In conclusion, our investigation provides evidence on the significance of shRNA targeting IL-6 in determining the tumorigenicity of pancreatic adenocarcinoma cells. We discovered that sensitivity of PANC-1 cells to gemcitabine could be enhanced by suppression of IL-6 with shRNA. Therefore, the combination of gemcitabine and shRNA targeting IL-6 may become a potential clinical application for the treatment of pancreatic adenocarcinoma.

## Figures and Tables

**Table 1:** Sequences of the primers for PCR.

Targets	Direction	Sequence
Bcl-2	Forward	GCCTTCTTTGAGTTCGGTG
	Reverse	AGTCATCCACAGGGCGAT
Bax	Forward	ATGGGCTGGACATTGGACTT
	Reverse	GCCACAAAGATGGTCACGGT
Caspase-3	Forward	ATCCAGTCGCTTTGTGCCAT
	Reverse	TTCTGTTGCCACCTTTCGGT
Caspase-9	Forward	TGGGCTCACTCTGAAGACCT
	Reverse	AGCAACCAGGCATCTGTTTA
JAK2	Forward	GCCTTCTTTCAGAGCCATCA
	Reverse	CCAGGGCACCTATCCTCATA
STAT3	Forward	AATACCATTGACCTGCCGATGT
	Reverse	GGTGGTCTCCTCTGACTTCAACA
GAPDH	Forward	GGTGGTCTCCTCTGACTTCAACA
	Reverse	GTTGCTGTAGCCAAATTCGTTGT
