# Corrigendum to “Suppression of IL-6 Gene by shRNA Augments Gemcitabine Chemosensitization in Pancreatic Adenocarcinoma Cells”

**DOI:** 10.1155/2022/9767389

**Published:** 2022-10-14

**Authors:** Hai-Bo Xing, Meng-Ting Tong, Jing Wang, Hong Hu, Chong-Ya Zhai, Chang-Xin Huang, Da Li

**Affiliations:** ^1^Department of ICU, Sir Run Run Shaw Hospital, School of Medicine, Zhejiang University, Hangzhou, China; ^2^Department of Medical Oncology, Sir Run Run Shaw Hospital, School of Medicine, Zhejiang University, Hangzhou, China; ^3^Department of Medical Oncology, The Affiliated Hospital of Hangzhou Normal University, Zhejiang University, Hangzhou, China

In the article titled “Suppression of IL-6 Gene by shRNA Augments Gemcitabine Chemosensitization in Pancreatic Adenocarcinoma Cells” [1], the reverse primer sequence for STAT3 given in Table 1 was incorrect. The authors have provided the correct primer sequence which is GGGTTCAGCACCTTCACCAT.

The corrected Table 1 is below.

## Figures and Tables

**Table 1:** Sequences of the primers for PCR.

Targets	Direction	Sequence
Bcl-2	Forward	GCCTTCTTTGAGTTCGGTG
	Reverse	AGTCATCCACAGGGCGAT

Bax	Forward	ATGGGCTGGACATTGGACTT
	Reverse	GCCACAAAGATGGTCACGGT

Caspase-3	Forward	ATCCAGTCGCTTTGTGCCAT
	Reverse	TTCTGTTGCCACCTTTCGGT

Caspase-9	Forward	TGGGCTCACTCTGAAGACCT
	Reverse	AGCAACCAGGCATCTGTTTA

JAK2	Forward	GCCTTCTTTCAGAGCCATCA
	Reverse	CCAGGGCACCTATCCTCATA

STAT3	Forward	AATACCATTGACCTGCCGATGT
	Reverse	GGGTTCAGCACCTTCACCAT

GAPDH	Forward	GGTGGTCTCCTCTGACTTCAACA
	Reverse	GTTGCTGTAGCCAAATTCGTTGT
